# Supplementary material for: Differences in levels of E. coli contamination of point of use drinking water in Bangladesh
Source: PLoS One. 2022 May 11;17(5):e0267386. doi: 10.1371/journal.pone.0267386 (PMC9094554; doi:10.1371/journal.pone.0267386)
Supplement: Attachment — Submitted filename: PONE-D-21-23710_review.pdf [file pone.0267386.s001.pdf]

PLOS ONE

# Inequalities in E-coli contamination in the point of use drinking water in Bangladesh

--Manuscript Draft--

| | |
|---|---|
| **Manuscript Number:** | PONE-D-21-23710 |
| **Article Type:** | Research Article |
| **Full Title:** | Inequalities in E-coli contamination in the point of use drinking water in Bangladesh |
| **Short Title:** | Inequalities in E-coli contamination in the point of use drinking water in Bangladesh |
| **Corresponding Author:** | Zahirul Hoque, Ph.D.<br>UAE University: United Arab Emirates University<br>Al Ain, UNITED ARAB EMIRATES |
| **Keywords:** | Drinking water, E-Coli, Bangladesh, Multinomial logistic regression. |
| **Abstract:** | The aim of this research is to provide empirical evidence to monitor the progress, to quantify the inequalities and to identify associated factors of the sustainable development goal (SDG) targets in relation to the safe drinking water. The level of E-Coli concentration in drinking water at the point of use (POU) and other information were extracted from the latest wave of the nationally representative Bangladesh Multiple Indicator Cluster Survey (MICS: 2019) data. The bivariate and multivariable multinomial logistic regression models and classification tree are used to find the specific combinations of the background characteristics with significantly higher rates of contamination.<br>A higher contamination risk of drinking water was observed if the household was categorised as middle or low wealth category and collects water from source with higher level of E-Coli concentration. Treatment of drinking water significantly reduces the risk of higher level of contamination whereas; having a pet was significantly associated with secondary contamination. Regional inequalities in the presence of E-Coli concentration in drinking water was evident.<br>The interventions in relation to the water source should emphasise on reducing the level of E-Coli contamination in the source water. Bangladesh must ensure safe drinking water source to the population in order to make further inroads towards reducing mortality relating to diarrheal diseases and improve overall contamination risks. |
| **Order of Authors:** | Zahirul Hoque, Ph.D. |
| | Md Masud Hasan |
| | Enamul Kabir |
| | Shahadut Hossain |
| **Additional Information:** | |
| **Question** | **Response** |
| **Financial Disclosure**<br><br>Enter a financial disclosure statement that describes the sources of funding for the work included in this submission. Review the submission guidelines for detailed requirements. View published research articles from *PLOS ONE* for specific examples.<br><br>This statement is required for submission and **will appear in the published article** if the submission is accepted. Please make sure it is accurate. | No funds, grants, or other supports were received. |

Powered by Editorial Manager® and ProduXion Manager® from Aries Systems Corporation

| | |
|---|---|
| **Unfunded studies**<br>Enter: *The author(s) received no specific funding for this work.*<br><br>**Funded studies**<br>Enter a statement with the following details:<br>• Initials of the authors who received each award<br>• Grant numbers awarded to each author<br>• The full name of each funder<br>• URL of each funder website<br>• Did the sponsors or funders play any role in the study design, data collection and analysis, decision to publish, or preparation of the manuscript?<br>• **NO** - Include this sentence at the end of your statement: *The funders had no role in study design, data collection and analysis, decision to publish, or preparation of the manuscript.*<br>• **YES** - Specify the role(s) played.<br><br>* typeset | |
| **Competing Interests**<br><br>Use the instructions below to enter a competing interest statement for this submission. On behalf of all authors, disclose any competing interests that could be perceived to bias this work—acknowledging all financial support and any other relevant financial or non-financial competing interests.<br><br>This statement is required for submission and **will appear in the published article** if the submission is accepted. Please make sure it is accurate and that any funding sources listed in your Funding Information later in the submission form are also declared in your Financial Disclosure statement.<br><br>View published research articles from *PLOS ONE* for specific examples. | The authors declare that they have no known competing financial interests or personal relationships which have, or could be perceived to have, influenced the work reported in this article. |

| | |
|---|---|
| **NO authors have competing interests**<br><br>Enter: *The authors have declared that no competing interests exist.*<br><br>**Authors with competing interests**<br><br>Enter competing interest details beginning with this statement:<br><br>*I have read the journal's policy and the authors of this manuscript have the following competing interests: [insert competing interests here]*<br><br>* typeset | |
| **Ethics Statement**<br><br>Enter an ethics statement for this submission. This statement is required if the study involved:<br><br>• Human participants<br>• Human specimens or tissue<br>• Vertebrate animals or cephalopods<br>• Vertebrate embryos or tissues<br>• Field research<br><br>Write "N/A" if the submission does not require an ethics statement.<br><br>General guidance is provided below. Consult the submission guidelines for detailed instructions. **Make sure that all information entered here is included in the Methods section of the manuscript.** | N/A |

| | |
|---|---|
| **Format for specific study types**<br><br>**Human Subject Research (involving human participants and/or tissue)**<br>• Give the name of the institutional review board or ethics committee that approved the study<br>• Include the approval number and/or a statement indicating approval of this research<br>• Indicate the form of consent obtained (written/oral) or the reason that consent was not obtained (e.g. the data were analyzed anonymously)<br><br>**Animal Research (involving vertebrate animals, embryos or tissues)**<br>• Provide the name of the Institutional Animal Care and Use Committee (IACUC) or other relevant ethics board that reviewed the study protocol, and indicate whether they approved this research or granted a formal waiver of ethical approval<br>• Include an approval number if one was obtained<br>• If the study involved *non-human primates*, add *additional details* about animal welfare and steps taken to ameliorate suffering<br>• If anesthesia, euthanasia, or any kind of animal sacrifice is part of the study, include briefly which substances and/or methods were applied<br><br>**Field Research**<br><br>Include the following details if this study involves the collection of plant, animal, or other materials from a natural setting:<br>• Field permit number<br>• Name of the institution or relevant body that granted permission | |
| **Data Availability**<br><br>Authors are required to make all data underlying the findings described fully available, without restriction, and from the time of publication. PLOS allows rare exceptions to address legal and ethical concerns. See the PLOS Data Policy and FAQ for detailed information. | Yes - all data are fully available without restriction |

| | |
|---|---|
| A Data Availability Statement describing where the data can be found is required at submission. Your answers to this question constitute the Data Availability Statement and **will be published in the article**, if accepted.<br><br>**Important:** Stating 'data available on request from the author' is not sufficient. If your data are only available upon request, select 'No' for the first question and explain your exceptional situation in the text box.<br><br>Do the authors confirm that all data underlying the findings described in their manuscript are fully available without restriction? | |
| **Describe where the data may be found in full sentences. If you are copying our sample text, replace any instances of XXX with the appropriate details.**<br><br>• If the data are **held or will be held in a public repository**, include URLs, accession numbers or DOIs. If this information will only be available after acceptance, indicate this by ticking the box below. For example: *All XXX files are available from the XXX database (accession number(s) XXX, XXX.)*.<br>• If the data are all contained **within the manuscript and/or Supporting Information files**, enter the following: *All relevant data are within the manuscript and its Supporting Information files.*<br>• If neither of these applies but you are able to provide **details of access elsewhere**, with or without limitations, please do so. For example:<br><br>*Data cannot be shared publicly because of [XXX]. Data are available from the XXX Institutional Data Access / Ethics Committee (contact via XXX) for researchers who meet the criteria for access to confidential data.*<br><br>*The data underlying the results presented in the study are available from (include the name of the third party* | UNICEF for Multiple Indicator Cluster Survey (www.childinfo.org) datasets. |

| | |
|---|---|
| *and contact information or URL).*<br>• This text is appropriate if the data are owned by a third party and authors do not have permission to share the data.<br><br>* typeset | |
| Additional data availability information: | |

Powered by Editorial Manager® and ProduXion Manager® from Aries Systems Corporation

Manuscript Click here to access/download;Manuscript;Manuscript.docx

# Inequalities in E-coli contamination in the point of use drinking water in Bangladesh

Md Masud Hasan[1], Zahirul Hoque[2,*], Enamul Kabir[3], Shahadat Hossain[4]

[1]Senior Research Fellow, Crawford School of Public Policy, ANU College of Asia and the Pacific, The Australian National University, Canberra, ACT 2601, Australia.

[2]United Arab Emirates University, Al Ain, UAE.

[3]University of Southern Queensland, Toowoomba, QLD 4350, Australia.

[4]Centre for Regulator Excellence, Statistics and Trials, Biologics and Radiopharmaceutical Drugs Directorate, Health Canada.

[*]Corresponding author:

Email: Zahirul.Hoque@uaeu.ac.ae

**Abstract**

The aim of this research is to provide empirical evidence to monitor the progress, to quantify the inequalities and to identify associated factors of the sustainable development goal (SDG) targets in relation to the safe drinking water. The level of E-Coli concentration in drinking water at the point of use (POU) and other information were extracted from the latest wave of the nationally representative Bangladesh Multiple Indicator Cluster Survey (MICS: 2019) [illegible]a. The bivariate and multivariable multinomial logistic regression models and classification tree are used to find the specific combinations of the background characteristics with significantly higher rates of contamination.

A higher contamination risk of drinking water was observed if the household was categorised as middle or low wealth category and collects water from source with higher level of E-Coli concentration. Treatment of drinking water significantly reduces the risk of higher level of contamination whereas; having a pet was significantly associated with secondary contamination. Regional inequalities in the presence of E-Coli concentration in drinking water was evident.

The interventions in relation to the water source should emphasise on reducing the level of E-Coli contamination in the source water. Bangladesh must ensure safe drinking water source to the population in order to make further inroads towards reducing mortality relating to diarrheal diseases and improve overall contamination risks.

Keywords: Drinking water, E-Coli, Bangladesh, Multinomial logistic regression.

## Introduction

The higher mortality rates from diarrheal diseases (in 2017, 1.6 m[illegible]on deaths globally including half a million children), predominantly from low- and middle-income countries can be substantially reduced through the interventions of safe drinking water[1]. Consuming safe drinking water is a human right [2] as reflected in the Millennium Development Goals (MDG) as ‘access to improved source’[3] and in the SDG as ‘safely managed’[4,5] targets. Contemporary studies showed that drinking water at the point of use (POU) is more likely to be contaminated than that at the main source and hence, the SDG targets beyond the infrastructure of source is more [illegible]tical and challenging[6,7]. The quality of drinking water in relation to health issues may be assessed through the presence or concentration of microbial contamination[8,9]. The SDG target of ‘safely managed’ water, in terms of free from microbial contamination, can be assessed using the presence or concentration of E-Coli (or other) microbes in drinking water. Based on the concentration of contamination, drinking water at source or at the POU are categorized with respect to potential health risks[10,11]. To monitor the achievements of safely managed drinking water in relation to the SDG targets and to develop related policy recommendations, contemporary literature aimed at determining the associated factors of E-Coli concentration in drinking water. One of the major sources of contamination is the collection point where drinking water may have exposed to germs from unhygienic environment. The sources are categorized as ‘improved’ or ‘unimproved’ based on their ability to supply safe drinking water through their construction or treatment[12]. Water collected from an ‘unimproved’ source is more likely to be contaminated during extraction, which may be carried on at the POU drinking water[10,13-15]. Type of main drinking water source and level of contamination at the collection point have an association with the level of contamination at the POU, though many of the improved sources may have faecal contamination above the WHO standard[16,17]. A higher level of faecal contamination in household water is associated with

unimproved sanitation facilities[18]. If households use open defecation or unimproved facilities, the germs from excreta can be exposed to water source, and consequently, quality of drinking water deteriorates. Secondary contamination of drinking water occurs due to poor management of household water resources, suc[illegible]s, unhygienic storage practice or water related behaviours which is not uncommon  in developing countries[19,20]. A clear understanding of the health impacts of consuming unsafe drinking water and safe handling practices to maintain quality of potable water can be achieved by passing information to individuals through educational institutions and/or community initiatives. A positive association between hygienic water practice and level of educational attainment is reported in the contemporary literature[21]. Increased risk of higher level of contamination of drinking-water at the point of consumption is significantly associated with ownership of livestock[22]. For example, a study with data from Ghana, Nepal and Bangladesh shows that ownership of any type of livestock was associated with an increased risk of faecal contamination of drinking water at the point of use[23,24]. Adequate water treatment methods at the point of use can significantly reduce the presence and total count of coliforms existed in the drinking water[25-27]. Socio-economic inequality among respondents was reflected in the access to quality livelihood, especially in terms of accessing potable water. A higher asset index score measured through household possessions was significantly associated with access to improved water sources and reduced E-coli contamination in the drinking water[28,29].

In literature, the general practice is to convert the E-Coli concentration of drinking water into the categories of potential health risks. The standard procedure of measuring the association of categorical outcome variable with a set of covariates is to measure the adjusted odds ratio in a multivariable analysis[30]. Based on the number of categories, a binary or trichotomous logistic regression models are fitted[10,31]. For three levels of ordered outcome variable measured as low, medium and high risks, an ordered logit model is preferred. The spatial correlation in the

distribution of the outcome variable is often incorporated using the Bayesian multivariable modelling framework[7].

This research aimed at understanding the distribution and inequalities in the E-Coli concentration in the point of use of drinking water at the households of Bangladesh. To accomplish the aim, association between E-Coli concentration and a set of covariates were measured. The [illegible]adjusted and adjusted effects were estimated using bivariate and multivariable analyses. A machine-learning tool, classification tree is used to identify the distribution of E-coli concentration over interactions of predictor variables. The analyses were conducted using the latest data of the nationally representative Multiple Indicator Cluster Survey (MICS) of Bangladesh conducted in 2019. The research has implications to better understand the SDG target 6.1 and to provide empirical evidence to support the development of feasible and effective plans to reach the target.

## Material and Methods

### Data

This study was conducted with the latest wave of the Multiple Indicator Cluster Survey conducted in Bangladesh in 2019 (MICS: 2019). The survey was designed to achieve reliable estimates at national level, across urban-rural areas, administrative divisions, and across districts of Bangladesh. A two-stage, stratified cluster sampling technique was adopted for the purpose of survey implementation. The first stage sampling frame was the primary sampling units (PSUs) obtained as the enumeration areas (EAs) based on the latest Bangladesh Population and Housing Census-2011. The main strata were defined as the urban and rural locations within each of the 64 districts.  A probability proportional to size (PPS) sampling procedure was used to select the PSUs (3220) from each of the sampling strata. For each of the selected EAs, complete list of households was prepared for the next stage sampling. A systematic random sample of 20 households was drawn from each of the 3220 EAs selected in first stage. From the selected 20 households from each EA, 4 households were selected for

assessing arsenic concentration in drinking water. From the four selected households of each of the EAs, two households were randomly sampled for assessing E-coli content in household drinking water and at the 'source' of drinking water. The sampling in these two stages were done using a systematic random sampling technique. Thus, the expected sample size of this study was 6,440 households those were selected for testing of E-coli. A 6,069 (98.7%) of the selected households were successfully tested for both household and source water quality for E-coli. Seventeen cases, for which results were lost or not readable, were excluded from the study.

## Variables

### Outcome variable

The dependent variable in this study is the quality of drinking water in terms of possible faecal contamination. The water samples were collected from the household by asking for 'a glass of water that you would give a child to drink'. The most recommended indicator for faecal contamination of drinking water in Bangladesh is the number of bacteria species Escherichia coli (E-coli) in a 100 ml sample of drinking water. The number of blue colonies as a measure of E-coli colony forming units (cfu) was recorded by MICS teams in the field. For this purpose, 100 ml of sample water is filtered through a 0.45 micron filter (Millipore Microfil®) and placed onto a Compact Dry EC growth media plates. After 24 hours of incubation at ambient temperature, number of blue colonies was recorded. The drinking water quality guidelines as recommended by the World Health Organisation (WHO) was followed to categorise recorded incubation into different risks levels. Household drinking water with less than one blue colony is termed as 'low risk', whereas, those within 1 to10 colonies were categorised as 'medium risk', and samples with 11 to 100 colonies and more than 100 colonies were categorised as 'high' and 'very high' risk, respectively. In this research, the last two categories are combined into the 'high risk category'.

### Predictor variables

In this study, a set of predictor variables were considered to test possible association with the outcome variable. The choice of the predictor variables was guided by the existing literature, knowledge of the researchers and availability of information.  The E-Coli contamination in the drinking water may be carried through the source of water collection. The information of this variable is recorded and categorized in the same way as the outcome variable. The type of drinking water sources (categorized as improved and unimproved) is considered as a potential predictor variable. The location of drinking water source may be linked to water quality in two ways, as they are located at areas surrounded by cleaner environment or may be contaminated through the carrying process. Based on the locations of drinking water sources, households were categorized as those having the sources in Dwelling/ Premises or elsewhere. The other predictor variables included into the analysis are whether the water was treated or untreated, type of toilet facility (improved and unimproved), place of residence and administrative division.  This research tested the hypothesis that educational attainment of household head influences the behavior of the household members towards consuming safe drinking water. Based on the educational attainment, household head were categorized as no education or pre-primary, primary and secondary level of education, or higher level of education. Several studies observed a positive association between ownership of livestock and contamination of water. With the ownership status of any livestock, herds, other farm animals, or poultry, households were categorized as either own or do not own any of the livestock. The study also tested whether the wealthy households, with better management, were able to keep the contamination to a lower level. Based on the household have higher abilities to manage safe drinking water, the variable was categorized as poor, middle or rich households.

**Statistical Analysis**

In specifying the determinants of E-Coli contamination in drinking water, the bivariate effects of the selected characteristics were examined. As the outcome variable as well as all covariates

are categorical by nature, bivariate $\chi^2$ analyses were carried out to identify the set of covariates with significant impact on the level of E-Coli contamination. However, bivariate association between two variables does not necessarily imply a significant relationship as this measure is not adjusted for other covariates. Therefore, a multivariable approac[illegible]as applied to determine the adjusted degrees of association between the covariates and outcome variable. The variable, E-coli contamination in household drinking water has three level and coded 2 (for high concentration), 1 (for moderate concentration) and 0 (for low concentration). For a multivariable analysis with three levels of outcome variable, a trich[illegible]mous logistic regression was employed and details of the model can be accessed through existing literature[32]. This is one of the mos[illegible]ble models to analyze outcome variables with three levels with meaningful and thorough interpretations[33,34]. This model has numerous applications in the domain of population health35,36. The trichotomous logistic regression model for modelling P(Y=1) and P(Y=2) as a function of a set of explanatory variables $(X_1, X_2, \dots, X_k)$ is given by

$$P(Y = 1) = \frac{e^{\boldsymbol{\beta}_1 X}}{1 + e^{\boldsymbol{\beta}_1 X + \boldsymbol{\beta}_2 X}}$$

$$P(Y = 2) = \frac{e^{\boldsymbol{\beta}_2 X}}{1 + e^{\boldsymbol{\beta}_1 X + \boldsymbol{\beta}_2 X}}$$

Where $\boldsymbol{\beta}_1$ is the set of regression coefficients associated with outcome 1, $\boldsymbol{\beta}_2$ is the set of regression coefficients associated with outcome 2; and X is the vector of explanatory variables, that is X=$(X_1, X_2, \dots, X_k$ ) where k is the number of explanatory variables.

In order to identify significant multifactor interactions of the covariates associated with the level of E-coli contamination, a classification tree method was used. The methodology is guided by the conditional inference framework[37,38]. The squared adjusted generalized variance inflation factor (GVIF) scores were used to quantify multicollinearity in the model[39]. The version 3.5.3 of open software 'R'[40] and related package "partykit" (version 1.2-7)[41] was utilized to analyze data to fit models.

**Results**

The results from bivariate analysis presenting the relationships between E-Coli contamination in drinking water and potential covariates are presented in **Error! Reference source not found.**. A higher level of contamination in source of drinking water resulted in a higher level of contamination at the point of use and the association was statistically significant ($p < 0.001$). Type of source of drinking water and location of the facility were significantly associated with the level of e-coli contamination at the POU. E-Coli contamination was significantly higher in water from unimproved sources than improved sources. The contamination was significantly lower in water collected from sources located in the household or premises than those located outside. The results from bivariate analysis also indicated that the proportion of households with e-coli contamination was lower for those using any water treatment methods. Percentages of households with a higher level of e-coli contamination was significantly lower in the household with improved toilet facilities. The ownership of livestock significantly increased the likelihood of consuming drinking water with possible faecal contamination. The education of household head showed a positive relationship with lower level of E-Coli contamination in drinking water. For example, the percentage of household with a low level of contamination was 15.5% to 21.4% when the head of the households had educational attainment were labelled as, ‘no education or pre-primary’ and ‘secondary or higher’ respectively.

Among the households categorized as poor, middle and rich, the proportion of sample households with a low level of E-Coli contamination were 15.0%, 17.4% and 21.4% respectively. A higher percentage of households from rural locations (62.8%) consumed water with high E-Coli concentration than that of urban households (56.8%). Percentages of households with high e-Coli concentration in drinking water was the highest in Barisal division followed by Dhaka division. The percentage was the lowest for Rangpur division followed by Mymenshing Division. The regional inequalities in the proportion of households with the level of E-Coli concentration was statistically significant.

The multivariable analysis using a trichotomous logistic regression was employed to quantify adjusted impacts of covariates on the E-coli contamination on drinking water with three levels. All the significant variables in the bivariate analysis were included in the model. Because of possible multi-collinearity of source and location of drinking water, the location of drinking water source is excluded from the model. The model outputs along with adjusted odds ratios (AOR) and 95% confidence intervals (CI) are presented in Table 2.

In the trichotomous logistic regression model, a higher level of E-coli concentration on the source of drinking water was significantly associated with the higher level of E-Coli concentration in the point of use drinking water. For example, households collecting water from a high-risk contaminated source were 11.92 (AOR: 12.92; 95% CI: 9.28 – 17.98) times more likely to have a high risk of E-coli contamination at the POU. On the other hand, for sources providing water with a moderate risk of contamination, the POU water were 5.32 (AOR: 6.32; 95% CI: 4.98 – 8.02) times more likely to have a high risk of E-Coli contamination at the POU. Households collecting drinking water from unimproved sources were more likely to consume water with a higher level of E-Coli contamination than that of collecting from improved sources. The relationship is statistically significant for a moderate risk of contamination (AOR: 2.51, 95% CI: 1.04 – 6.03).

The households not treating their drinking water after collection were significantly more likely to consume water with moderate ($p < 0.05$, AOR: 1.44; 95% CI: 1.04 – 1.99) and high risk ($p < 0.001$, AOR: 1.80; 95% CI: 1.35 – 2.40) of E-coli contamination. Ownership of pet was significantly associated ($p < 0.001$) with the consumption of water with a higher level of E-coli contamination. Considering the households without a livestock, those owned, were 0.35 (AOR: 1.35; 95% CI: 1.15 – 1.58) times more likely to consume drinking water labelled as high risks of contamination. The other variable related to household environment was the type of toilet facility used in the household. Not possessing a toilet facility or using an unimproved one was

related to a higher risk of E-Coli concentration in the drinking water. However, the association was not statistically significant.

Wealth of household held a consistent association with the level of E-Coli concentration risks in the potable water, though the relationship was more evident when considering the contamination related to a higher risk. For example, with respect to the rich household, the middle and poor households, respectively, were 0.43 (AOR: 1.43; 95% CI: 1.13 – 1.81) and 0.74 times (AOR: 1.74; 95% CI: 1.33 – 2.26) more likely to consume water with a high risk of E-coli contamination. Urban households were less likely to have E-Coli contaminated drinking water at the POU, though the differences were not statistically significant. This study identifies significant regional inequalities in E-Coli contaminated water at the point from where the household members drink. Households residing in Barisal and Khulna divisions were, respectively, 3.41 (AOR: 4.41; 95% CI: 3.10 – 6.26) times and 1.06 (AOR: 2.06; 95% CI: 1.57 – 2.70) times more likely to use high E-Coli contaminated drinking water compared to those residing at Rangpur division.

Levels of contamination risks related to E-Coli concentration in drinking water at POU in Bangladesh for various combinations of the levels of background of household is presented in Figure 1. The root node of the classification tree is the level of E-Coli concentration in the source of water. This finding indicates that contamination of water at the source is the major contributor in the contamination risks in POU drinking water. Urban-rural residence does not appear to be a significant contributor to the outcome variable when the households collected drinking water with high level of E-Coli concentration. The immediate left of the trunk is divided into branches based on the wealth of the household. For households consuming water from high-contaminated source, the contamination level at POU drinking water did not differ significantly between the households with poor or middle income. For this group, the highest percentages of households (91.0%) had a high level of E-coli concentration in the POU

drinking water (node 6). The third node (second node to the left trunk) indicates significant regional inequalities in the level of E-Coli concentration (amongst the households categorised as rich and using drinking water from sources with high level of E-Coli concentration). The right trunk from the root node refers to households collecting drinking water with low or medium level of concentration. All the terminal nodes through this trunk (nodes 9, 11, 14, 15, 16, 18, 20 and 21) are related to relatively lower percentages of households with high level of E-coli concentration. From this group, relatively higher proportion of households (67.0%) from Barishal divisions had higher level of E-Coli contamination in drinking water (node 9). The proportion is followed (66.4%; node 15) by the poor households in Dhaka and Khulna divisions and middle/poor households from Rajshahi divisions (62.1%; node 16).

**Discussion**

This research monitors the progress, quantifies the inequalities and identifies the associated factors of the SDG targets in the safe 'point of use' drinking water in Bangladesh in terms of microbiological quality. A largely used indicator, concentration of E-Coli, is adopted to determine microbiological quality[42] of water. The inequalities in E-Coli concentration over the levels of a set of covariates is first identified through bivariate analyses. Results from the bivariate analysis are not adjusted for other variables, and hence multivariate analyses are conducted. The trichotomous logistic regression model, a multivariate approach, is applied to determine which factors best explain the E-Coli contamination in the drinking water. Finally, the machine-learning tool, classification tree is used to identify multifactor interactions of significance between the predictors of E-Coli contaminated drinking water. This manuscript used data from the latest wave of the nationally representative Multiple Indicator Cluster Survey (MICS) of Bangladesh conducted in 2019.

The source of drinking water did not have significant association with the high level of E-Coli concentration in the POU water. This research indicates that using any water treatment methods and E-Coli concentration in the POU drinking water had a negative association. These findings

explains the facts that improved source of drinking water was not significantly associated with the incidence of childhood diarrhea in Bangladesh, India and Pakistan[43]. However, experiencing multiple health conditions among the children in Bangladesh had significant association with the combination of the access to improved drinking water source and treatment of the drinking water[44].

This study identifies that a higher level of E-Coli concentration in the water source was significantly associated with the level of E-Coli concentration in the POU drinking water. The fact is supported by a study in Rohingya camps, Banglade[illegible]. The positive association between proportion of households with high level of E-Coli concentration in the POU water and having a pet may indicates the possibilities of secondary contamination. Middle/poor households with high level of E-Coli concentration at source water possess approximately 90% of risks of having a high level contamination at the POU water.

The results from this study suggests a higher proportion of E-Coli contamination were originated from the source of drinking water. Emphasis should put on improving the infrastructure of water source to ensure that water is not being contaminated through surrounding environment. A large proportion of water from ‘improved’ sources were observed as contaminated. The traditional definition of ‘improved water source’ should be revised and physical environment of the facility should also include to determine the status of the facility. Integrated plans should be taken through the central and local Government and NGOs to provide [illegible]quate water treatment facilities to the mass population. Formal and informal educational programmes should put in place to educate people regarding the water hygiene. The awareness programs should include the risks of secondary contamination by cattle or through improper storage systems. Significant regional inequalities in microbial contamination demands the adaptation of alternative approaches at local level. Bangladesh must ensure safe

drinking water source to the population in order to make further inroads towards reducing mortality relating to diarrheal diseases and improve overall contamination risks.

## Conclusion

Like other low- and middle-income countries, microbial contamination of drinking water is a major public health concern in Bangladesh. Using multivariable statistical models and machine learning tools, to the latest nationally representative survey in Bangladesh, this study identified associated factors of E-Coli concentration at the POU drinking water.

Despite nearly universal access of ‘improved’ sources, E-Coli concentration at the POU drinking water was identified as a major public health concern in Bangladesh. This contamination was significantly associated with the contamination of water at the source but not with the type (improved or unimproved) of the source. Hence, key causes of contamination of water at the source of collection should be identified and measures should be taken to avoid such contamination. Secondary contamination of drinking water was significantly associated with the ownership of livestock. The rural households should be educated regarding the possible secondary contamination of drinking water through livestock. Use of water treatment facilities significantly reduces the e-coli contamination of drinking water, though the use is limited. Integrated campaign regarding the importance of treating water before drinking may excel the current rates of water treatment users. The potential water treatment users should also educate how to use the methods effectively to make water safe and the materials should be readily available. Significant regional inequalities in the presence of E-Coli concentration in POU of source of drinking water should be kept in mind in developing relevant policies. Integrated policies incorporating research informed measures would help achieving the SDG’s ‘safely managed’ water targets.

***Acknowledgments:*** UNICEF for Multiple Indicator Cluster Survey (www.childinfo.org) datasets.

***Ethical approval:*** Ethical approval for the analyses was not sought as the paper is based on de-identified data.

***Declaration of interest:*** This research does not contain any conflict of interest.

Table 1: Percentage distribution of households with various level of E-coli contaminations in drinking water at the point of use.

| | E Coli in POU drinking water | | | Sample size (N) |
|---|---|---|---|---|
| | Low | Moderate | High | |
| *E-Coli in water source ($p < 0.001$)* | | | | |
| Low | 25.9 | 23.3 | 50.8 | 3741 |
| Moderate | 6.6 | 21.3 | 72.0 | 1326 |
| High | 4.4 | 6.8 | 88.8 | 985 |
| *Education of household head ($p < 0.001$)* | | | | |
| No or pre primary | 15.5 | 19.6 | 65.0 | 2142 |
| Primary | 17.4 | 19.4 | 63.1 | 1714 |
| Secondary or higher | 21.4 | 21.4 | 57.2 | 2196 |
| *Ownership of livestock ($p < 0.001$)* | | | | |
| No | 21.4 | 20.6 | 58.0 | 2354 |
| Yes | 16.1 | 19.9 | 64.0 | 3698 |
| *Household wealth status ($p < 0.001$)* | | | | |
| Poor | 15.0 | 19.7 | 65.3 | 2796 |
| Middle | 18.8 | 20.1 | 61.1 | 2350 |
| Rich | 26.3 | 21.9 | 51.9 | 906 |
| *Source of drinking water ($p < 0.001$)* | | | | |
| Improved | 18.6 | 20.3 | 61.1 | 5877 |
| Unimproved | 4.0 | 15.4 | 80.6 | 175 |
| *Location of drinking water source ($p < 0.001$)* | | | | |
| Dwelling/ Premises | 19.3 | 20.7 | 60.0 | 4373 |
| Elsewhere | 12.1 | 19.2 | 68.7 | 1260 |
| *Treatment of drinking water ($p = 0.002$)* | | | | |
| No | 17.6 | 20.2 | 62.1 | 5558 |
| Yes | 24.3 | 19.4 | 56.3 | 494 |
| *Type of toilet facility ($p < 0.001$)* | | | | |
| Improved | 19.0 | 20.5 | 60.5 | 5027 |
| Unimproved | 14.3 | 18.5 | 67.1 | 1025 |
| *Place of residence ($p < 0.001$)* | | | | |
| Rural | 17.1 | 20.1 | 62.8 | 4896 |
| Urban | 22.8 | 20.3 | 56.8 | 1156 |
| *Administrative division ($p < 0.001$)* | | | | |
| Barisal | 9.3 | 21.5 | 69.2 | 558 |
| Chittagong | 18.3 | 22.3 | 59.5 | 1046 |
| Dhaka | 16.9 | 16.6 | 66.6 | 1232 |
| Khulna | 15.3 | 19.9 | 64.8 | 947 |
| Mymenshing | 21.9 | 24.3 | 53.8 | 370 |
| Rajshahi | 21.9 | 15.4 | 62.7 | 764 |
| Rangpur | 24.5 | 24.8 | 50.7 | 747 |
| Sylhet | 18.8 | 21.4 | 59.8 | 388 |

Table 2: Adjusted odds ratio, confidence interval and p-value of moderate and high risk of E-coli contamination of drinking water at the point of use obtained from the multinomial logistic regression models.

| | Moderate risk | | High risk | |
|---|---|---|---|---|
| | AOR (LCL – UCL) | P-value | AOR (LCL –UCL) | P-value |
| (Intercept) | 0.44 (0.29 – 0.67) | 0.000 | 0.37 (0.25 – 0.53) | 0.000 |
| E-Coli in water source | | | | |
| Low | 1.00 | --- | 1.00 | --- |
| Moderate | 3.79 (2.92 – 4.92) | 0.000 | 6.32 (4.98 – 8.02) | 0.000 |
| High | 1.80 (1.19 – 2.71) | 0.005 | 12.92 (9.28 – 17.98) | 0.000 |
| Ownership of livestock | | | | |
| No | 1.00 | --- | 1.00 | --- |
| Yes | 1.17 (0.97 – 1.40) | 0.102 | 1.35 (1.15 – 1.58) | 0.000 |
| Source of drinking water | | | | |
| Improved | 1.00 | --- | 1.00 | --- |
| Unimproved | 2.51 (1.04 – 6.03) | 0.043 | 1.70 (0.75 – 3.85) | 0.206 |
| Treatment of drinking water | | | | |
| Yes | 1.00 | --- | 1.00 | --- |
| No | 1.44 (1.04 – 1.99) | 0.026 | 1.80 (1.35 – 2.40) | 0.000 |
| Education of Household head | | | | |
| Secondary or above | | | | |
| No/Pre primary | 1.10 (0.89 – 1.37) | 0.370 | 1.18(0.98 – 1.42) | 0.083 |
| Primary | 0.98 (0.79 – 1.22) | 0.887 | 1.09(0.90 – 1.31) | 0.377 |
| Type of toilet facility | | | | |
| Improved | 1.00 | --- | 1.00 | --- |
| Unimproved | 1.01 (0.79 – 1.30) | 0.865 | 1.14 (0.92 – 1.42) | 0.214 |
| Household wealth status | | | | |
| Rich | 1.00 | --- | 1.00 | --- |
| Middle | 1.14 (0.87 – 1.49) | 0.335 | 1.43 (1.13 – 1.81) | 0.003 |
| Poor | 1.28 (0.95 – 1.73) | 0.106 | 1.74 (1.33 – 2.26) | 0.000 |
| Place of residence | | | | |
| Rural | 1.00 | --- | 1.00 | --- |
| Urban | 0.91 (0.72 – 1.15) | 0.429 | 0.85 (0.70 – 1.04) | 0.124 |
| Administrative division | | | | |
| Rangpur | 1.00 | --- | 1.00 | --- |
| Barisal | 2.55 (1.73 – 3.77) | 0.000 | 4.41 (3.10 – 6.26) | 0.000 |
| Chittagong | 1.16 (0.86 – 1.56) | 0.310 | 1.21 (0.93 – 1.59) | 0.154 |
| Dhaka | 1.03 (0.76 – 1.38) | 0.820 | 1.82 (1.41 – 2.40) | 0.000 |
| Khulna | 1.24 (0.91 – 1.69) | 0.164 | 2.06 (1.57 – 2.70) | 0.000 |
| Mymenshing | 0.97 (0.67 – 1.41) | 0.941 | 0.87 (0.62 – 1.22) | 0.418 |
| Rajshahi | 0.75 (0.54 – 1.03) | 0.073 | 1.54 (1.18 – 2.00) | 0.001 |
| Sylhet | 1.25 (0.84 – 1.84) | 0.255 | 1.59 (1.13 – 2.25) | 0.008 |

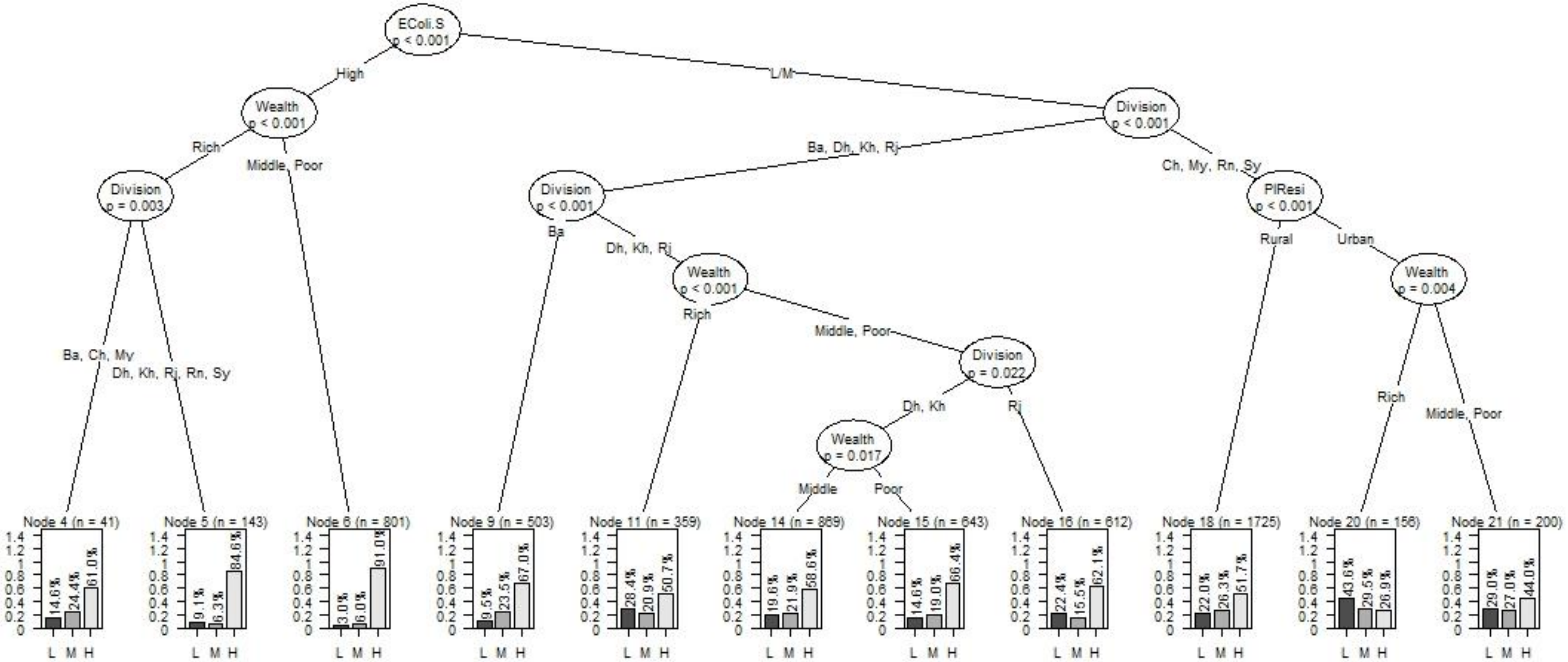


Figure 1: Classification tree representing the distribution of the level of E-Coli concentration of drinking water over the combinations of the levels of the household characteristics

List of Tables:

Table 2: Percentage distribution of households with various level of E-coli contaminations in drinking water at the point of use.

Table 2: Adjusted odds ratio, confidence interval and p-value of moderate and high risk of E-coli contamination of drinking water at the point of use obtained from the multinomial logistic regression models.

List of Figures:

Figure 2 Classification tree representing the distribution of the level of E-Coli concentration of drinking water over the combinations of the levels of the household characteristics.

References:


1. Roser M, Ritchie H, Dadonaite B. Child and infant mortality. Our World in Data. In:2019.
2. Assembly UG. Resolution 70/169: The Human Rights to Safe Drinking Water and Sanitation. In:2015.
3. R Grafton Q, Wyrwoll P, White C, Allendes D. *Global Water Issues and Insights.* ANU Press; 2014.
4. Weststrate J, Dijkstra G, Eshuis J, Gianoli A, Rusca M. The sustainable development goal on water and sanitation: learning from the millennium development goals. *Social Indicators Research.* 2019;143(2):795-810.
5. Schmidt-Traub G, De la Mothe Karoubi E, Espey J. *Indicators and a Monitoring Framework for the Sustainable Development Goals: Launching a Data Revolution for the SDGs.* Sustainable Development Solutions Network; 2015.
6. Khan SM, Bain RE, Lunze K, et al. Optimizing household survey methods to monitor the Sustainable Development Goals targets 6.1 and 6.2 on drinking water, sanitation and hygiene: A mixed-methods field-test in Belize. *PloS one.* 2017;12(12):e0189089.
7. Khan JR, Bakar KS. Spatial risk distribution and determinants of E. coli contamination in household drinking water: a case study of Bangladesh. *International Journal of Environmental Health Research.* 2020;30(3):268-283.
8. Osmani M, Mali S, Hoxha B, Bekteshi L, Karamelo P, Gega N. Drinking water quality determination through the water pollution indicators, Elbasan district. *Thalassia Salentina.* 2019;41:3-10.
9. Wu D, Wang H, Seidu R. Smart data driven quality prediction for urban water source management. *Future Generation Computer Systems.* 2020;107:418-432.
10. Kandel P, Kunwar R, Lamichhane P, Karki S. Extent of fecal contamination of household drinking water in Nepal: Further analysis of Nepal Multiple Indicator Cluster Survey 2014. *The American journal of tropical medicine and hygiene.* 2017;96(2):446-448.
11. Kusmawati W, Rahayu L. Contamination of Escherichia coli Drinking Water Refills on Drinking Water Depots in Malang City. *Biogenesis: Jurnal Ilmiah Biologi.* 2019;7(1):9-13.
12. UNICEF, WHO. Progress on Drinking Water, Sanitation and Hygiene: 2017 Update and SDG Baselines. *World Health Organization (WHO) and the United Nations Children's Fund (UNICEF) Geneva.* 2017.
13. Lyimo B, Buza J, Subbiah M, Smith W, Call DR. Comparison of antibiotic resistant Escherichia coli obtained from drinking water sources in northern Tanzania: a cross-sectional study. *BMC microbiology.* 2016;16(1):254.
14. Harris AR, Pickering AJ, Boehm AB, Mrisho M, Davis J. Comparison of analytical techniques to explain variability in stored drinking water quality and microbial hand contamination of female caregivers in Tanzania. *Environmental Science: Processes & Impacts.* 2019;21(5):893-903.
15. Osiemo MM, Ogendi GM, M'Erimba C. Microbial quality of drinking water and prevalence of water-related diseases in Marigat Urban Centre, Kenya. *Environmental health insights.* 2019;13:1178630219836988.
16. Dekker DM, Krumkamp R, Sarpong N, et al. Drinking water from dug wells in rural Ghana—Salmonella contamination, environmental factors, and genotypes. *International journal of environmental research and public health.* 2015;12(4):3535-3546.
17. Bain R, Cronk R, Wright J, Yang H, Slaymaker T, Bartram J. Fecal contamination of drinking-water in low- and middle-income countries: a systematic review and meta-analysis. *PLoS Med.* 2014;11(5):e1001644.
18. Kundu A, Smith WA, Harvey D, Wuertz S. Drinking water safety: Role of hand hygiene, sanitation facility, and water system in semi-urban areas of India. *The American Journal of Tropical Medicine and Hygiene.* 2018;99(4):889-898.
19. Shaheed A, Orgill J, Montgomery MA, Jeuland MA, Brown J. Why? improved? water sources are not always safe. *Bulletin of the World Health Organization.* 2014;92:283-289.
20. Mahmud ZH, Islam MS, Imran KM, et al. Occurrence of Escherichia coli and faecal coliforms in drinking water at source and household point-of-use in Rohingya camps, Bangladesh. *Gut pathogens.* 2019;11(1):1-11.
21. Uwimpuhwe M, Reddy P, Barratt G, Bux F. The impact of hygiene and localised treatment on the quality of drinking water in Masaka, Rwanda. *Journal of Environmental Science and Health, Part A.* 2014;49(4):434-440.
22. Ercumen A, Pickering AJ, Kwong LH, et al. Animal feces contribute to domestic fecal contamination: evidence from E. coli measured in water, hands, food, flies, and soil in Bangladesh. *Environmental science & technology.* 2017;51(15):8725-8734.

23. Wardrop NA, Hill AG, Dzodzomenyo M, Aryeetey G, Wright JA. Livestock ownership and microbial contamination of drinking-water: Evidence from nationally representative household surveys in Ghana, Nepal and Bangladesh. *International journal of hygiene and environmental health.* 2018;221(1):33-40.
24. Schriewer A, Odagiri M, Wuertz S, et al. Human and animal fecal contamination of community water sources, stored drinking water and hands in rural India measured with validated microbial source tracking assays. *The American journal of tropical medicine and hygiene.* 2015;93(3):509-516.
25. De Kwaadsteniet M, Dobrowsky P, Van Deventer A, Khan W, Cloete T. Domestic rainwater harvesting: microbial and chemical water quality and point-of-use treatment systems. *Water, Air, & Soil Pollution.* 2013;224(7):1629.
26. Usman MA, Gerber N, von Braun J. The impact of drinking water quality and sanitation on child health: Evidence from rural Ethiopia. *The Journal of Development Studies.* 2019;55(10):2193-2211.
27. Edokpayi JN, Rogawski ET, Kahler DM, et al. Challenges to sustainable safe drinking water: a case study of water quality and use across seasons in rural communities in Limpopo province, South Africa. *Water.* 2018;10(2):159.
28. Opryszko MC, Guo Y, MacDonald L, MacDonald L, Kiihl S, Schwab KJ. Impact of water-vending kiosks and hygiene education on household drinking water quality in rural Ghana. *The American journal of tropical medicine and hygiene.* 2013;88(4):651-660.
29. Hasan MM, Alam K. Inequality in access to improved drinking water sources and childhood diarrhoea in low-and middle-income countries. *International Journal of Hygiene and Environmental Health.* 2020;226:113493.
30. Diaz AA, Tomba E, Lennarson R, Richard R, Bagajewicz MJ, Harrison RG. Prediction of protein solubility in Escherichia coli using logistic regression. *Biotechnology and bioengineering.* 2010;105(2):374-383.
31. Invik J, Barkema HW, Massolo A, Neumann NF, Cey E, Checkley S. Escherichia coli contamination of rural well water in Alberta, Canada is associated with soil properties, density of livestock and precipitation. *Canadian Water Resources Journal/Revue canadienne des ressources hydriques.* 2019;44(3):248-262.
32. Hosmer DW, Lemeshow S. *Applied Logistic Regression.* John Wiley and Sons, Inc; 1989.
33. Hosmer DW, Lemeshow S, Sturdivant RX. *Applied logistic regression.* Vol 398: John Wiley & Sons; 2013.
34. Harrell FJ. *Regression modelling strategies: with applications to linear models, logistic and ordinal regression, and survival analysis.* Springer; 2015.
35. Park H. An introduction to logistic regression: from basic concepts to interpretation with particular attention to nursing domain. *Journal of Korean Academy of Nursing.* 2013;43(2):154-164.
36. Austin PC, Steyerberg EW. Graphical assessment of internal and external calibration of logistic regression models by using loess smoothers. *Statistics in medicine.* 2014;33(3):517-535.
37. James G, Witten D, Hastie T, Tibshirani R. *An introduction to statistical learning.* Vol 112: Springer; 2013.
38. Hothorn T, Hornik K, Zeileis A. Unbiased recursive partitioning: A conditional inference framework. *Journal of Computational and Graphical statistics.* 2006;15(3):651-674.
39. Fox J, Monette G. Generalized collinearity diagnostics. *Journal of the American Statistical Association.* 1992;87(417):178-183.
40. Team RC. R version 3.5. 3: A language and environment for statistical computing. *R Foundation for Statistical Computing, Vienna, Austria URL https://www R-project org.* 2019.
41. Hothorn T, Hornik K, Zeileis A. ctree: Conditional inference trees. *The Comprehensive R Archive Network.* 2015;8.
42. Odonkor ST, Ampofo JK. Escherichia coli as an indicator of bacteriological quality of water: an overview. *Microbiology research.* 2013;4(1):e2-e2.
43. Hasan MM, Richardson A. How sustainable household environment and knowledge of healthy practices relate to childhood morbidity in South Asia: analysis of survey data from Bangladesh, Nepal and Pakistan. *BMJ open.* 2017;7(6):e015019.
44. Kamal MM, Hasan MM, Davey R. Determinants of childhood morbidity in Bangladesh: evidence from the demographic and health survey 2011. *BMJ open.* 2015;5(10):e007538.
